# Elements of End-of-Life Discussions Associated With Patients’ Reported Outcomes and Actual End-of-Life Care in Patients With Pretreated Lung Cancer

**DOI:** 10.1093/oncolo/oyad245

**Published:** 2023-09-05

**Authors:** Takaaki Hasegawa, Toru Okuyama, Takehiro Uemura, Yoshinobu Matsuda, Hiroyuki Otani, Junichi Shimizu, Yoshitsugu Horio, Naohiro Watanabe, Teppei Yamaguchi, Satoshi Fukuda, Tetsuya Oguri, Ken Maeno, Yoshihiko Taniguchi, Kaname Nosaki, Kensuke Fukumitsu, Tatsuo Akechi

**Affiliations:** Center for Psycho-oncology and Palliative Care, Nagoya City University Hospital, Nagoya, Aichi, Japan; Center for Psycho-oncology and Palliative Care, Nagoya City University Hospital, Nagoya, Aichi, Japan; Department of Psychiatry and Cognitive-Behavioral Medicine, Nagoya City University Graduate School of Medical Sciences, Nagoya, Aichi, Japan; Department of Psychiatry/Palliative Care Center, Nagoya City University West Medical Center, Nagoya, Aichi, Japan; Department of Respiratory Medicine, Allergy and Clinical Immunology, Nagoya City University Graduate School of Medical Sciences, Nagoya, Aichi, Japan; Department of Thoracic Oncology, Aichi Cancer Center Hospital, Nagoya, Aichi, Japan; Department of Psychosomatic Internal Medicine, National Hospital Organization, Kinki-Chuo Chest Medical Center, Sakai, Osaka, Japan; Department of Palliative Care Team, Palliative and Supportive Care, National Hospital Organization Kyushu Cancer Center, Fukuoka, Fukuoka, Japan; Department of Palliative Care Team, Palliative and Supportive Care, St. Mary’s Hospital, Kurume, Fukuoka, Japan; Department of Thoracic Oncology, Aichi Cancer Center Hospital, Nagoya, Aichi, Japan; Department of Thoracic Oncology, Aichi Cancer Center Hospital, Nagoya, Aichi, Japan; Department of Thoracic Oncology, Aichi Cancer Center Hospital, Nagoya, Aichi, Japan; Department of Thoracic Oncology, Aichi Cancer Center Hospital, Nagoya, Aichi, Japan; Department of Respiratory Medicine, Allergy and Clinical Immunology, Nagoya City University Graduate School of Medical Sciences, Nagoya, Aichi, Japan; Department of Respiratory Medicine, Allergy and Clinical Immunology, Nagoya City University Graduate School of Medical Sciences, Nagoya, Aichi, Japan; Department of Education and Research Center for Community Medicine, Nagoya City University Graduate School of Medical Sciences, Nagoya, Aichi, Japan; Department of Respiratory Medicine, Allergy and Clinical Immunology, Nagoya City University Graduate School of Medical Sciences, Nagoya, Aichi, Japan; Department of Internal Medicine, National Hospital Organization, Kinki-Chuo Chest Medical Center, Sakai, Osaka, Japan; Department of Thoracic Oncology, National Hospital Organization Kyushu Cancer Center, Fukuoka, Fukuoka, Japan; Department of Thoracic Oncology, National Cancer Center Hospital East, Kashiwa, Chiba, Japan; Department of Respiratory Medicine, Allergy and Clinical Immunology, Nagoya City University Graduate School of Medical Sciences, Nagoya, Aichi, Japan; Center for Psycho-oncology and Palliative Care, Nagoya City University Hospital, Nagoya, Aichi, Japan; Department of Psychiatry and Cognitive-Behavioral Medicine, Nagoya City University Graduate School of Medical Sciences, Nagoya, Aichi, Japan

**Keywords:** neoplasm, lung cancer, end-of-life discussion, communication, advance care planning, palliative care

## Abstract

**Background:**

End-of-life discussions for patients with advanced cancer are internationally recommended to ensure consistency of end-of-life care with patients’ values. This study examined the elements of end-of-life discussions associated with end-of-life care.

**Materials and Methods:**

We performed a prospective observational study among consecutive patients with pretreated non-small cell lung cancer after the failure of first-line chemotherapy. We asked oncologists whether they had ever discussed “prognosis,” “do not attempt resuscitation,” “hospice,” and “preferred place of death” with a patient at baseline. The quality of life (QOL) and depressive symptoms of patients were assessed using validated questionnaires at baseline and 3 months later. The end-of-life care that patients received was investigated using medical records. Oncologists’ compassion and caregivers’ preferences for hospice care were also assessed using questionnaires. Multiple regression analyses were conducted to examine the association between elements of end-of-life discussions and patient-reported outcomes as well as actual end-of-life care.

**Results:**

We obtained 200 valid responses at baseline, 147 valid responses 3 months later, and 145 data points for medical care at the end-of-life stage. No element of the end-of-life discussion between the patient and their oncologist was significantly associated with patients’ reported outcomes or actual end-of-life care. In addition, oncologists’ compassion was significantly associated with improvement in both comprehensive QOL and depressive symptoms, and caregivers’ preferences for hospice care and high educational level were significantly associated with hospice death.

**Conclusion:**

Oncologist-patient alliances and caregivers’ involvement in end-of-life discussions may be influential in achieving optimal end-of-life care.

## Teaser

This study evaluated elements of end-of-life discussion on comprehensive quality of life and depressive symptoms in patients with incurable non-small cell lung cancer, as well as which elements are most associated with care decisions and the role of oncologists’ empathy and caregivers’ preferences in such decisions.

**Implications for Practice**

None of the elements of the end-of-life discussions between patients with pretreated non-small cell lung cancer and their oncologists were significantly associated with patients’ reported outcomes, including quality of life (QOL) and depressive symptoms, or actual end-of-life care, such as hospice death. Oncologists’ compassion was significantly associated with improvement in both comprehensive QOL and fewer depressive symptoms, and caregivers’ preferences for hospice care and high educational level were significantly associated with hospice death. Oncologist-patient alliances and caregivers’ involvement in end-of-life discussions may be influential in achieving optimal end-of-life care.

## Introduction

The value of end-of-life discussions and advance care planning have been increasingly recognized in enhancing the quality of end-of-life care.^1^ The purpose of end-of-life discussions is to ensure that the patient receives medically appropriate care consistent with their values.^2,3^ End-of-life discussions can reduce aggressive end-of-life care, which is associated with an even worse quality of life (QOL).^4^ In a previous systematic review, end-of-life discussions were defined as any conversation about the goal of end-of-life care or treatment preferences with a healthcare provider or trained facilitator, documented in medical records or self-reported by patients, surrogates, or documentation.^5^ End-of-life discussions do not address a single topic, but address multiple related to the goals of end-of-life care.^6,7^ Among them, important elements include discussions related to prognosis, preferred site of death, hospice care, and not attempting resuscitation in patients with advanced cancer.^8,9^ Generally, end-of-life discussions in patients with cancer take place near the end-of-life in the illness trajectory and often during hospital admission.^10^

Evidence of the effectiveness of end-of-life discussions on patients’ QOL is limited.^11^ We are not aware of a study that has investigated what elements of end-of-life discussions are associated with comprehensive QOL. This study seeks to evaluate the effects of each element of end-of-life discussion on comprehensive QOL in patients with incurable non-small cell lung cancer (NSCLC), with a life expectancy of ≤1 year. Oncologists are often concerned that end-of-life discussions may increase psychological distress in patients.

Thus, this study also evaluates the effects of each element of end-of-life discussions on patients’ depressive symptoms. It also investigates what elements of end-of-life discussions are associated with end-of-life care, including the place of death and aggressive end-of-life care.^12,13^ In addition, increased attention has been paid toward the patient-oncologist relationship^14,15^ and the disagreement between patient and caregiver preferences for end-of-life care.^16^ Thus, we also examine whether oncologists’ empathy and caregivers’ preferences for end-of-life care affect it.

## Materials and Methods

### Study design

This study is part of a longitudinal study focusing on prognostic awareness that we had initially planned and had previously reported.^17^ The details of the study design have been described elsewhere. Briefly, we prospectively studied patients with advanced or postoperative NSCLC recurrence at 4 sites in Japan. Consecutive patients who attended medical follow-up appointments with the participating oncologists were screened. The institutional review board at each site approved the study protocol. The study was conducted in accordance with the principles of the Declaration of Helsinki. All patients were provided with a detailed description of the purpose and methods of the study before completing the questionnaire. Written informed consent was obtained from all the patients. The study was registered with the University Hospital Medical Information Network (UMIN000026436).

### Patients

Patients were included if they were (1) diagnosed with stage IIIB not amenable to curative treatment, stage IV, or postoperative recurrent NSCLC; (2) aged ≥20 years; (3) at the stage within 2 months after the failure of first-line chemotherapy; and (4) able to understand written or spoken Japanese. The exclusion criteria were as follows: (1) too ill to complete the questionnaire, (2) severe mental disorders, (3) severe cognitive disorders, or (4) judgment by the treating oncologist as unsuitable for participation. We also included patients with NSCLC with targetable genetic aberrations whose disease progressed after chemotherapy and whose prior therapy included molecular-targeted therapy.

### Measurement

#### Elements of End-of-Life Discussion Reported by Oncologists

To assess the status of end-of-life discussion, we asked oncologists if they had ever discussed the following with a patient: “prognosis,” “do not attempt resuscitation,” “hospice,” and “preferred place of death.”^8-10^ Response options for each of these questions were “yes” or “no.”

#### Patients’ Reported Outcomes

##### Quality of Life

We measured the comprehensive QOL of patients using the Comprehensive QOL Outcome (CoQoLo) inventory,^18^ which is a validated and reliable tool for measuring QOL based on the concept of good death in patients with advanced cancer. We used a short version of the CoQoLo. This scale is capable of comprehensively measuring QOL outcomes independent of patients’ general physical condition. Higher values indicated better comprehensive QOL.

##### Depressive Symptoms

Depressive symptoms were measured using the Patient Health Questionnaire-9 (PHQ-9).^19^ Higher values indicated more severe depressive symptoms. The PHQ-9 has been validated in a Japanese population.^20^

#### Actual End-of-Life Care

##### Place of Death

Place of death was categorized into home hospice, acute care unit (cancer hospital), inpatient hospice/palliative care unit, and long-term care hospital.

##### Intensity of End-of-Life Care

High-intensity end-of-life (HI-EOL) care consisted of the following: ≥one session of IV chemotherapy in <14 days from death, starting a new IV chemotherapy regimen in <30 days from death, ≥one hospitalization in an intensive care unit (ICU) during the final 30 days of life, >one emergency room admission during the final 30 days of life, or >one hospitalization in an acute care unit during the final 30 days of life.^21^ Most invasive end-of-life (MI-EOL) care consisted of the following: death in the ICU, intubation and/or ventilation during the final 30 days of life, or cardiopulmonary resuscitation during the final 30 days of life. We defined intensive end-of-life care as HI-EOL and/or MI-EOL care.

#### Preferred Place of End-of-Life Care

We asked patients, “At end-of-life care, where would you rather get treatment when you have a difficulty in transportation to visit outpatient medical facilities.” The response options were “home hospice,” “hospital,” or “inpatients hospice/palliative care unit.” Patients and caregivers were asked to complete the questionnaire at baseline.

Participants who chose “home hospice” or “inpatients hospice/palliative care unit” were coded as “preferring hospice care.” Participants who chose “hospital” were coded as “preferring hospital care.” We measured both patients’ and caregivers’ preferred places of end-of-life care as independent factors in exploring the association between hospice death and end-of-life discussions. This is because in East Asian countries, including Japan, there is a tendency to prioritize the wishes of the caregiver over those of the patient, known as family-centered decision-making.^22^

##### Oncologist Compassion

Oncologist compassion was measured using the Physician Compassion Questionnaire (PCQ). This scale comprises 5 dimensions: warm/cold, pleasant/unpleasant, compassionate/distant, sensitive/insensitive, and caring/uncaring. Each dimension is scored on a scale of 0 to 10. The sum of the 5 scales yields a final score of 0-50 points. Lower scores indicate greater physician compassion (0 = best, 50 = worst).^23,24^ We measured oncologist compassion assuming that it is associated with communication between the oncologist and the patient, which relates to patients’ psychological distress and QOL.^14,15,25^

##### Perceptions of Incurability

We asked patients, “How would you describe your current health condition?” The response options were “good, curable”; “serious, but curable”; “good, but incurable”; “serious, incurable”; “I don’t know”; or “I don’t wish to answer,” with reference to previous studies.^26,27^ Patients who chose “good, but incurable” or “serious, incurable” were designated as having “accurate perception of incurability.” Patients who responded “good, curable”; “serious, but curable”; “I don’t know”; or “I don’t wish to answer” were designated as “inaccurate perception of incurability.”

### Data Collection

Patients completed a baseline questionnaire, including demographic characteristics, CoQoLo, PHQ-9, PCQ, perceptions of incurability, and preferred place of end-of-life care, within 2 months after progressive disease of first-line chemotherapy. We administered follow-up assessments to them 3 months later (or at a clinic visit within 4 weeks of that time). Follow-up assessments included the CoQoLo and PHQ-9. The caregivers who participated in this study were identified as primary caregivers, and they completed a baseline questionnaire that included demographic characteristics and their preferred place of end-of-life care. The researchers contacted participants in case they found unanswered data. We obtained patient characteristics, including age and sex, HI-EOL care, MI-EOL care, and place of death, from medical records. Performance status and lung cancer histology were obtained from oncologists at baseline.

### Statistics

The analyses began with descriptive summaries of the demographic and clinical variables. In addition, we summarized the reported end-of-life discussions.

To investigate whether each element of end-of-life discussions was associated with changes in patient-reported outcomes (CoQoLo or PHQ-9) from baseline to 3 months later, a multiple logistic regression analysis was performed after adjusting for age, education, baseline score (CoQoLo or PHQ-9), oncologist compassion (PCQ score), and perception of incurability (accurate/inaccurate).^14,15,25^ In addition, the association between each element of end-of-life discussions and place of death (inpatient or home hospices/acute care unit) was examined using multiple logistic regression analysis adjusted for age, education, oncologist compassion (PCQ score), perception of incurability (accurate/inaccurate), and preferred place of end-of-life care (patient and caregiver preferences).^28^ The association between the intensity of end-of-life care and end-of-life discussions could not be evaluated because only a small number of patients received intensive end-of-life care.

Statistical significance was set at *P* < .05. Statistical analyses were performed using SPSS v.28.0 (IBM Corp., Armonk, NY, USA).

## Results

### Flow Diagram and Characteristics of Patients and Caregivers

**Figure 1.**
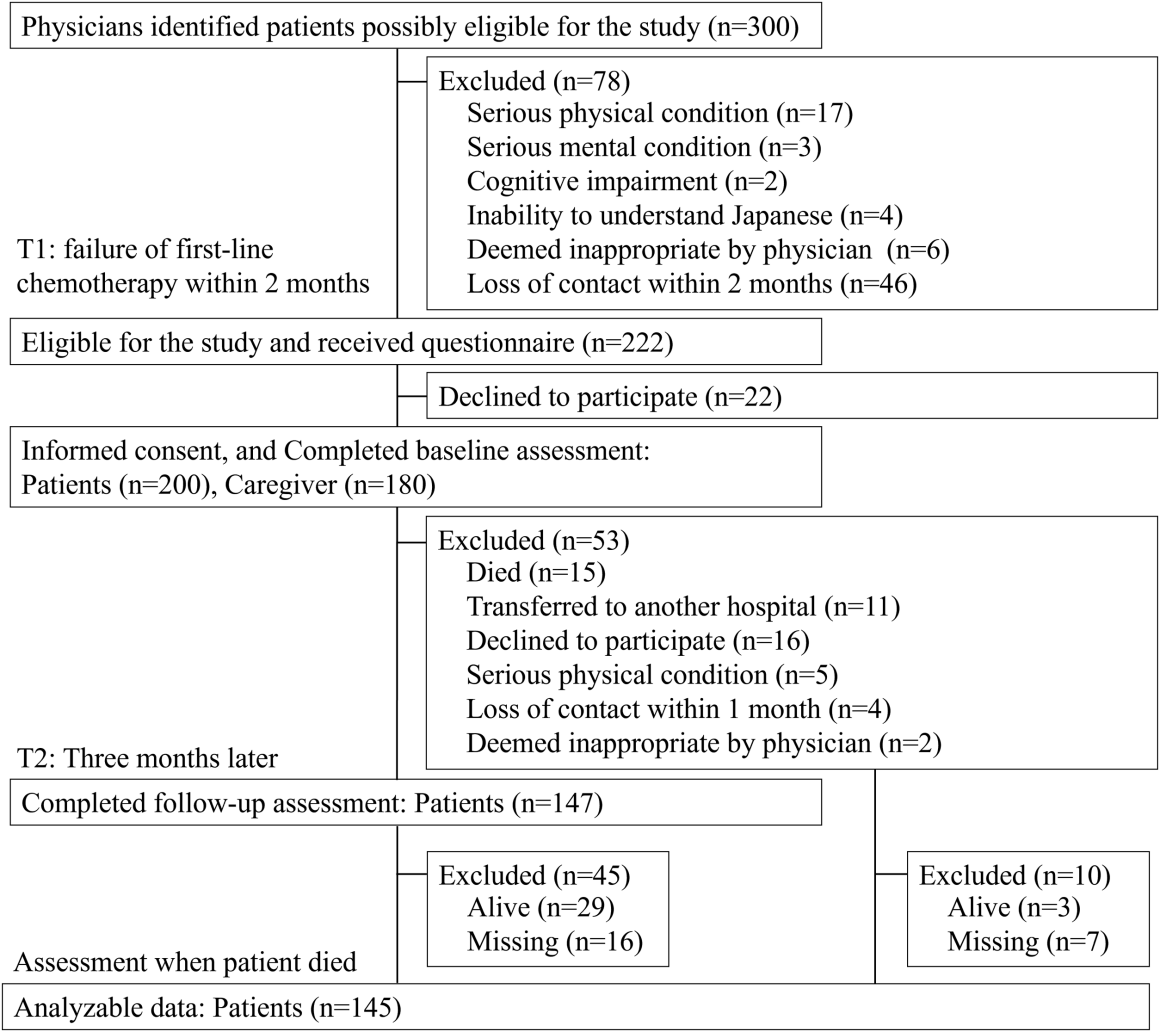
Flow chart showing recruitment of participants and follow-up.

During the study period, 300 potential participants were identified, 222 of whom were eligible and 200 returned valid questionnaire responses (Fig. 1). All patients who participated received anticancer treatment at baseline. Valid responses were obtained from 147 patients after 3 months. Table 1 summarizes participants’ characteristics. The place of death and intensity of end-of-life care were obtained from 145 patients. The median (95% confidence interval [CI]) overall survival was 254 days (range = 221-287).

**Table 1.** Patient and caregiver characteristics.

	Patients			Caregivers
Characteristic	Baseline (within 2 months after first-line failure), *n* = 200	3 months later, *n* = 147	Deceased during study period, *n* = 145	Baseline (within 2 months after first-line failure), *n* = 180
	Total *n* (%)	Total *n* (%)	Total *n* (%)	Total *n* (%)
Age (years)				
Mean (SD)	65.1 (10.0)	65.4 (9.9)	66.1 (9.7)	58.8 (13.2)
Sex				
Male	137 (68.5)	102 (69.4)	101 (69.7)	63 (35.0)
Female	63 (31.5)	45 (30.6)	44 (30.3)	116 (64.4)
Marital status				
Married	158 (79.0)	121 (82.3)	115 (79.3)	Not assessed
Other	42 (21.0)	26 (17.7)	30 (20.7)	Not assessed
Relationship with patient				
Spouse	Not applicable	Not applicable	Not applicable	125 (69.4)
Child	Not applicable	Not applicable	Not applicable	34 (18.9)
Parent	Not applicable	Not applicable	Not applicable	8 (4.4)
Sibling	Not applicable	Not applicable	Not applicable	8 (4.4)
Other	Not applicable	Not applicable	Not applicable	5 (2.8)
Education				
≥High school	161 (80.5)	124 (84.4)	112 (77.2)	160 (88.9)
<High school	38 (19.0)	23 (15.6)	32 (22.1)	19 (10.6)
Occupation				
Paid or self-employed (full-time)	31 (15.5)	25 (17.0)	23(15.9)	46 (25.6)
Paid employee (part-time)	15 (7.5)	12 (8.2)	11 (7.6)	33 (18.3)
Homemaker	29 (14.5)	21 (14.3)	18 (12.4)	59 (32.8)
Retirement	100 (50.0)	77 (52.4)	74 (51.0)	30 (16.7)
Other	24 (12.0)	12 (8.2)	18 (12.4)	12 (6.7)
Living condition				
Living with someone	175 (87.5)	131 (89.1)	126 (86.9)	Not assessed
Living alone	25 (12.5)	16 (10.9)	19 (13.1)	Not assessed
PS				
0	72 (36.0)	62 (42.2)	45 (31.0)	Not applicable
1	117 (58.5)	81 (55.1)	91 (62.8)	Not applicable
2	11 (5.5)	4 (2.7)	9 (6.2)	Not applicable
Histology				
Non-Sq	150 (75.0)	112 (76.2)	108 (74.5)	Not applicable
Sq	50 (25.0)	35 (23.8)	37 (25.5)	Not applicable

Some items did not reach the total number due to missing values.

Abbreviations: PS: Eastern Cooperative Oncology Group performance status; Sq: squamous cell carcinoma.

### Frequency of Each Element of End-of-Life Discussion

There were wide variations in the frequency of each element of end-of-life discussions: “prognosis” (23.5%) was the most frequently reported, followed by “do not attempt resuscitation (9.0%),” “hospice (8.5%),” and “preferred place of death (3.5%).”

### Association Between Each Element of End-of-Life Discussions and Patient-Reported Outcomes (QOL and Depression)

The mean (±SD) CoQoLo score was 52.9 (±7.6) at baseline and 54.4 (±8.6) at 3 months. The mean (±SD) PHQ-9 score was 6.2 (±4.8) at baseline and 5.2 (±4.2) at 3 months. The mean (± SD) PCQ score was 12.8 (±9.8) at baseline.

Multiple regression analysis revealed that end-of-life discussions and the perception of incurability were not associated with changes in CoQoLo or PHQ-9 scores (Table 2). However, an improvement in the CoQoLo score was significantly associated with greater oncologist compassion and younger age (<75 years). Worsening of the PHQ-9 score was significantly associated with low oncologist compassion.

**Table 2.** Association between each element of end-of-life discussions and patient-reported outcomes (QOL and depression) or hospice deaths.

	Improvement of CoQoLo score, *n* = 147		Worsen of PHQ-9 score, *n* = 147		Death at hospice, *n* = 145	
	β-coefficients (95% CI)	*P*	β-coefficients (95% CI)	*P*	Adjusted odds ratio (95% CI)	*P*
Characteristic						
EOLd (prognosis)	−0.08 (−3.92 to 1.44)	.36	0.08 (−0.69 to 2.34)	.28	1.05 (0.41 to 2.66)	.93
EOLd (DNAR)	−0.10 (−8.05 to 2.37)	.28	0.11 (−0.86 to 4.94)	.17	1.95 (0.36 to 10.69)	.44
EOLd (hospice)	−0.06 (−8.26 to 4.98)	.62	0.03 (−2.86 to 3.79)	.78	0.18 (0.02 to 1.90)	.15
EOLd (place of death)	0.01 (−8.06 to 9.02)	.91	−0.03 (−5.34 to 4.14)	.80	0.70 (0.03 to 18.90)	.83
Oncologist compassion	−0.33 (−0.35 to −0.11)	<.001	0.23 (0.03 to 0.17)	.004	1.01 (0.97 to 1.05)	.72
Perception of incurability (accurate)	−0.03 (−2.64 to 1.84)	.73	0.04 (−0.96 to 1.62)	.62	1.18 (0.52 to 2.68)	.69
Patient’s preference for hospice care	-	-	-	-	0.98 (0.40 to 2.40)	.95
Caregiver’s preference for hospice care	-	-	-	-	2.54 (1.04 to 6.19)	.041
Age (≥75 years)	−0.20 (−6.56 to −0.64)	.017	0.12 (−0.32 to 3.09)	.11	2.33 (0.82 to 6.60)	.11
Education (≥ high school)	−0.05 (−3.92 to 2.15)	.57	-0.04 (−2.24 to 1.22)	.56	6.28 (2.06 to 19.15)	.001
CoQoLo score	−0.44 (−0.55 to −0.24)	<.001	-	-	-	-
PHQ-9 score	-	-	−0.67 (−0.76 to −0.49)	<.001	-	-

Abbreviations: QOL: quality of life; EOLd: end-of-life discussion; DNAR: do not attempt resuscitation; CoQoLo: Comprehensive Quality of Life Outcome; PHQ-9: Patient Health Questionnaire-9.

### Association Between Each Element of End-of-Life Discussions and Place of Death

Place of death data are presented in Table 3 (ICU death data are presented in Table 4): 46.9% (95% CI, 38.6%-55.4%) of patients died in inpatient hospices or home hospices and 35.2% (95% CI, 27.4%-43.5%) died in the acute care unit of a cancer hospital.

**Table 3.** Place of death.

Place of death	*n*	%
Acute care unit (cancer hospital)	51	35.2
Home hospice	38	26.2
Inpatient hospice/palliative care unit	30	20.7
Long-term care hospital	21	14.5
Other	5	3.4

**Table 4.** Intensity of end-of-life care during the final 30 days of life.

Intense and invasive care	*n*	%
High-intensity end-of-life care	27	18.6
Intravenous chemotherapy within 14 days before death	3	2.1
Starting a new IV chemotherapy regimen within 30 days before death	9	6.2
>One emergency room visit within 30 days before death	4	2.8
>One hospitalization	18	12.4
ICU admission within 30 days before death	1	0.7
Most invasive end-of-life care	3	2.1
ICU death	1	0.7
Intubation and/or ventilation within 30 days before death	2	1.4
Cardiopulmonary resuscitation within 30 days before death	3	2.1

Abbreviation: ICU: intensive care unit.

Multiple logistic regression analysis revealed that no element of end-of-life discussion was associated with hospice death (home hospice or inpatient hospice/palliative care unit; Table 2). However, caregivers’ preference for hospice care at baseline and patient’s education level (≥high school) were significantly associated with death at hospice (home hospice or inpatient hospice/palliative care unit).

### Intensity of End-of-Life Care

The intensity of the end-of-life treatment is shown in Table 4. Among the 145 deceased patients, 18.6% (95% CI, 12.6%-25.9%) underwent HI-EOL care and 2.1% (95% CI, 0.2%-4.9%) underwent MI-EOL care. We could not evaluate the association between the intensity of end-of-life care and end-of-life discussions because only a small number of patients received HI-EOL and/or MI-EOL care.

### Additional Findings

Preferred places of end-of-life care are presented in Table 5. A total of 137 (68.5%) patients (95% CI, 61.6-74.9) and 110 (61.1%) caregivers preferred hospice care at baseline (95% CI, 53.6-68.3).

**Table 5.** Place preferred for end-of-life care.

	Patients	Caregivers
Preferred place of end-of-life care	Baseline, *n* = 200 *n* (%)	Baseline, *n* = 180 *n* (%)
Acute care unit (cancer hospital)	61 (30.5)	65 (36.1)
Home hospice	83 (41.5)	54 (30.0)
Inpatient hospice/palliative care unit	54 (27.0)	56 (31.1)

The agreement between patients’ and caregivers’ preferred place of end-of-life care at baseline was 56%.

## Discussion

To the best of our knowledge, this is the first prospective cohort study to examine how different aspects of end-of-life discussions relate to patient-reported outcomes, including QOL (representing important aspects of a good death) and depressive symptoms in patients with advanced or postoperative recurrent NSCLC.

This study had several strengths. First, prior studies have recommended advance care planning for patients similar to those included in this study (life expectancy of ≤1 year, tumor progression after prior therapy, etc.).^1^ Second, the study cohort comprised consecutive eligible patients at multiple study sites, and the rates of refusal and missing data for medical care near death were within acceptable limits.

The first major finding was that none of the elements of end-of-life discussions were associated with QOL based on the concept of good death. In a previous scoping review, advance care planning intervention for patients with advanced cancer had no effect on the generic QOL.^7,29^ Advance care planning interventions are designed to ensure that patients receive care that is consistent with their goals.^2,3,30^ Its most appropriate goal is not to improve QOL. The end-of-life discussions measured in this study may not have directly improved QOL based on the concept of good death. Alternatively, the timing of the measurement of QOL (at 3 months after first-line chemotherapy failure) might have been too early to determine whether each element of the end-of-life discussion was effective for QOL.^31^ The possibility remains that end-of-life discussions could be effective for improving the QOL just before death (called the quality of death and dying). The finding that end-of-life discussions were not associated with the worsening of depressive symptoms is also consistent with a previous scoping review.^29^ It appears that oncologists do not have to avoid end-of-life discussions out of a concern for worsening patients’ depressive symptoms. Perception of incurability was not associated with QOL based on the concept of good death and depressive symptoms.

The second major finding was that end-of-life discussions about the preferred place of death were not associated with death in hospice care. The association between hospice use and end-of-life discussions remains controversial as in the previous reports.^11^ A finding in one previous cohort study (Cancer Care Outcomes Research and Surveillance [CanCORS]), found that patients who had end-of-life discussions were more likely to receive hospice care than those who did not have these discussions.^32^ This inconsistency between this study and CanCORs could be owing to various factors. First, this study had fewer end-of-life discussions with oncologists, and the identification of end-of-life discussions in this study occurred earlier than that in the CanCORS study. Second, the low rate of invasive end-of-life care in Japan could have influenced our findings. The impact of end-of-life discussions on end-of-life care may not be observed because aggressive end-of-life care is rarely offered.^33^ Third, cultural differences must be considered, as families have a greater influence on decision-making. Our study revealed that death during hospice care was not associated with patient preference for hospice care but was associated with caregiver preference. Thus, end-of-life discussions between patients and their oncologists may not affect the place of death in Japan. Traditionally, family-centered decision-making has been conducted in East Asian countries.^34,35^ However, the importance of caregiver involvement in decision-making is being recognized internationally, not only in Asia; and how to involve caregivers is an important issue.^36,37^ Another finding was that less-educated patients were less likely to receive hospice care; that is, less-educated patients might need to be supported in decision-making.

Of note, greater oncologist compassion was significantly associated with an improved QOL. Moreover, low oncologist compassion was significantly associated with worsening depressive symptoms. These findings are consistent with those of a previous study reporting that oncologists’ compassion has the potential to alleviate patients’ psychological distress.^23^ These findings suggest the importance of enhancing the care of patients at the moment, rather than talking about future end-of-life care.^38^ Alternatively, an empathetic oncologist could provide psychological care or symptom management to alleviate patients’ psychological distress.

This study had several limitations. First, the questionnaire used to assess the preferred place for end-of-life care was not fully validated. However, face validity was confirmed in a pilot test involving 5 patients with cancer. Second, this is a prospective cohort study and not an interventional trial. It documents the presence of elements in end-of-life discussions without evaluating their quality. Third, this study was conducted at 4 institutions. Our findings cannot be generalized to other settings such as other types and clinical stages of cancer. Fourth, there could have been recall bias in the questionnaires used to evaluate the provision of end-of-life discussions between patients and their oncologists. Fifth, building upon existing research, this study focuses on 4 elements that are deemed foundational among these diverse components. However, it is widely recognized that end-of-life discussions encompass a broad range of variations. It is important to acknowledge the existence of key aspects that are not addressed in this study, such as engaging in conversations regarding patients’ goals and values and symptom management.^39^

## Conclusion

Our findings suggest that none of the elements of end-of-life discussions are associated with the quality of end-of-life care, including QOL, based on the concepts of good death, depressive symptoms, and place of death. Further research is needed to examine the importance of oncologists having empathetic discussions with their patients, along with their caregivers.

## Data Availability

The data underlying this article will be shared on reasonable request to the corresponding author.
